# The polymorphisms of MIR31HG gene is correlated with alcohol-induced osteonecrosis of the femoral head in Chinese Han male population

**DOI:** 10.3389/fendo.2022.976165

**Published:** 2022-11-24

**Authors:** Wei Liu, Xin Wang, JianFei Chen, Fan Zeng, Jun Xiong

**Affiliations:** Department of Orthopedic Trauma, Hainan General Hospital, Hainan Affiliated Hospital of Hainan Medical University, Haikou, China

**Keywords:** osteonecrosis of the femoral head, MIR31HG, polymorphism, case-control study, gene

## Abstract

**Background:**

Alcoholic osteonecrosis of the femoral head (ONFH) is a multifaceted illness that seriously disturbs the patients’ quality of life. The role of lncRNAs in alcoholic ONFH has attracted widespread attention in recent years. This study mainly explored whether MIR31HG polymorphism affects the risk of ONFH.

**Methods:**

There were 733 males (308 alcohol-induced ONFH patients and 425 healthy controls). Seven single nucleotide polymorphisms from MIR31HG were genotyped using the Agena MassARRAY platform. Odds ratio (OR) and 95% confidence intervals (CI) *via* logistic regression was applied to assess the contribution of MIR31HG variants to alcoholic ONFH susceptibility.

**Results:**

We found that rs10965059 was related to a lower risk of alcoholic ONFH in the overall, age, and necrotic sites analysis. Rs10965064 also showed a risk-reducing effect in the occurrence of alcoholic ONFH patients older than 40 years old.

**Conclusions:**

We confirmed that MIR31HG variants have a significant correlation with the occurrence of alcoholic ONFH among the Chinese Han male population. our findings may provide new ideas for understanding the effect of MIR31HG on the prevention and diagnosis of alcoholic ONFH.

## Introduction

Osteonecrosis of the femoral head (ONFH) is a common hip illness characterized by impaired microvascular circulation leading to the death of bone cells. It eventually causes structural changes, collapse of the femoral head, and joint dysfunction. It is reported that the age of patients with ONFH is mostly 30~50 years old, and the incidence rate is gradually increasing with the increase of age (1). There are 8.12 million ONFH patients in China, with approximately 150,000 to 200,000 newly diagnosed ONFH patients yearly (2). Some research showed that one of the common causes of ONFH was excessive drinking (3, 4). Besides, Cui et al. reported that alcoholic ONFH accounted for 30.7% of all femoral head necrosis in China (5). Nevertheless, it has been found in clinical practice that only some people who drink excessively suffer from femoral head necrosis, which suggested that genetic susceptibility may contribute to the occurrence of alcoholic ONFH. Meanwhile, a large number of reports confirmed that genetic variants were related to alcoholic ONFH predisposition (6–8).

Long non-coding RNAs (lncRNAs) refers to non-coding RNA with a length of over 200 nucleotides, which can regulate the physiological and pathological activities of organisms by participating in gene transcription and post-transcriptional regulation, epigenetic modification, and translation (9, 10). Recently, lncRNAs have aroused researcher’s concern in bone development, differentiation, and osteonecrosis. For example, Tang et al. found that lncRNA-OG could promote the differentiation of osteoblasts (11). Liu et al. indicated that lncRNA AK077216 facilitated bone resorption and osteoclastogenesis (12). Besides, a study performed by Xiang et al, which reported that lncRNA RP11-154D6 was reduced in ONFH patients and involved in the progression of ONFH (13).

MIR31HG (also named as lncHIFCAR, LOC554202) is described to participant in proliferation, differentiation, invasion of cancer cells (14, 15). In addition, researchers have discovered that MIR31HG silence promoted osteogenic differentiation and relieved the inflammation-induced inhibition of osteogenesis (16). However, the role of MIR31HG in alcoholic ONFH development remains elusive. However, the role of MIR31HG in alcoholic ONFH development remains elusive.

Here, we explored the association of MIR31HG genetic variations with alcoholic ONFH susceptibility among the Chinese Han male population. This will help to provide new understandings for MIR31HG into the pathogenesis of alcoholic ONFH.

## Methods

### Study subjects

A total of 733 Chinese Han males (308 alcoholic ONFH patients and 425 healthy controls) were enrolled. All patients were randomly selected from Hainan Affiliated Hospital of Hainan Medical University. The patients met the criteria as following: 1) exceeded 400 mL/week (17) alcohol intake for more than 6 months; 2) diagnosed ONFH within one year after drinking alcohol; 3) no rheumatoid arthritis, hyperlipidemia, spinal cord cavitation, decompression sickness, osteoporosis, cardiovascular disease, and virus infection; 4) no history of steroid use; 5) The diagnosis of alcoholic ONFH by X-ray, nuclear magnetic resonance imaging (MRI), and computed tomography (CT). The stage of alcoholic ONFH patients were identified by the Ficat Classification system (18). Healthy people were selected based on 1) drinking habits or greater than 400 mL alcohol intake per week for more than 6 months; 2) no history of the traumatic disease (ONFH, rheumatoid arthritis, hyperlipidemia, spinal cord cavitation, decompression sickness, osteoporosis, cardiovascular disease, etc…), and no steroid use. The study protocol was approved by the Ethics Committee of the Hainan Affiliated Hospital of Hainan Medical University, in compliance with the declaration of Helsinki. And the signature consent of participants was received.

### SNP genotyping

Seven SNPs in MIR31HG were chosen with a minor allele frequency (MAF) >5% in 1000 Genomes Chinese Han Beijing population. Total DNA was isolated from peripheral blood cells by GoldMag blood DNA Kit (GoldMag Co. Ltd, Xi’an, China), and the concentration were evaluated *via* NanoDrop 2000 (Thermo Scientific, USA). SNP genotyping was completed through the Agena MassARRAY platform and the data were analyzed using the Agena Typer 4.0 software.

### Data analysis

Student t-test was applied to evaluate age difference between the two groups. Hardy-Weinberg equilibrium (HWE) in controls was calculated using *χ* ^2^ test. The linkage between MIR31HG polymorphisms and alcoholic ONFH susceptibility was examined by odds ratio (OR) and 95% confidence interval (CI) through logistic regression. Linkage disequilibrium (LD) was analyzed by Haploview software. Multifactor dimensionality reduction (MDR) was conducted to evaluate the SNP-SNP interactions in the risk of ONFH. False positive report probability (FPRP) values and statistical power were calculated (19). *P* < 0.05 was identified a significant difference.

## Results

### Participants and selected SNPs in MIR31HG

As listed in **Table 1**, this study included 308 alcoholic ONFH patients and 425 healthy controls, with an average age of 43.37 ± 11.34 years and 42.73 ± 12.88 years, respectively. The age distribution between the two groups was well-matched (*p*=0.478).

**Table 1:** Characteristics of ONFH patients and controls in this study.

Variables	Cases (n=308)	Controls (n=425)	*p* value
Age, years	43.37 ± 11.34	42.73 ± 12.88	0.478
≤40	187 (61%)	246 (58%)	
>40	121 (39%)	179 (42%)	
Clinical stages			
I-II	218 (71%)		
III-IV	90 (29%)		
Hip lesions			
Unilateral	65 (21%)		
Bilateral	243 (79%)		
Course, months			
>22	103 (33%)		
≤22	205 (67%)		

*p* value was calculated from student’s t test.

We chose and genotyped seven SNPs (rs1332184, rs72703442, rs2025327, rs55683539, rs2181559, rs10965059, rs10965064) in the intron region of MIR31HG, and all SNPs in line with HWE (*p*>0.05). And we found that rs10965059 could reduce the susceptibility of alcoholic ONFH in the allele model (*p* < 0.001, OR = 0.48, 95%CI=0.35-0.66, **Table 2**).

**Table 2:** Basic information of the selected SNPs in MIR31HG.

SNP	Chr : Position	Role	AlleleA/B	MAF		HWE*p*	OR (95% CI)	*p*	HaploReg
				Case	Control			
rs1332184	9:21504203	Intron	A/G	0.291	0.259	0.256	1.17 (0.93-1.48)	0.178	Enhancer histone marks, DNAse
rs72703442	9:21515795	Intron	A/C	0.172	0.160	0.858	1.09 (0.82-1.44)	0.552	Enhancer histone marks, Motifs changed
rs2025327	9:21531629	Intron	C/T	0.141	0.114	1.000	1.28 (0.94-1.74)	0.122	Enhancer histone marks, DNAse, Motifs changed, Selected eQTL hits
rs55683539	9:21542134	Intron	T/C	0.244	0.245	0.089	0.99 (0.78-1.26)	0.938	Enhancer histone marks, DNAse, Motifs changed, Proteins bound
rs2181559	9:21543938	Intron	A/T	0.378	0.352	0.340	1.12 (0.90-1.39)	0.298	Enhancer histone marks, DNAse, Motifs changed, Proteins bound, Selected eQTL hits
rs10965059	9:21544062	Intron	T/C	0.096	0.181	0.070	0.48 (0.35-0.66)	**<0.001**	DNAse, Motifs changed, Proteins bound
rs10965064	9:21553538	Intron	G/C	0.356	0.369	0.678	0.94 (0.76-1.17)	0.585	DNAse, Motifs changed

Human (GRCh38.p13) reference is used for SNP annotation.

SNP, Single nucleotide polymorphism; MAF, Minor allele frequency; HWE, Hardy-Weinberg equilibrium; OR, Odds ratio; 95% CI, 95% confidence interval.

*p* values were calculated from χ^2^ test. Bold values indicate statistical significance.

### Alcoholic ONFH risk assessment

The association of seven SNPs in MIR31HG and alcoholic ONFH risk was evaluated (**Table 3**). The results revealed that MIR31HG- rs10965059 was related to a lower risk of alcoholic ONFH under codominant (*p*<0.001, OR=0.43, 95%CI=0.30-0.62), dominant (*p*<0.001, OR=0.42, 95%CI=0.30-0.61) and additive models (*p*<0.001, OR=0.45, 95%CI=0.32-0.62).

**Table 3:** Association of MIR31HG polymorphisms with alcohol-induced ONFH.

SNP ID	Model	Genotype	Case	Control	Crude analysis		Adjusted analysis	
					OR (95% CI)	*p*	OR (95% CI)	*p*
rs1332184	Codominant	GG	159	238	1.00		1.00	
		AA	30	33	1.36 (0.80-2.32)	0.258	1.38 (0.81-2.35)	0.240
		AG	119	154	1.16 (0.85-1.58)	0.361	1.15 (0.84-1.57)	0.377
	Dominant	GG	159	238	1.00		1.00	
		AA+AG	149	187	1.19 (0.89-1.60)	0.241	1.19 (0.89-1.60)	0.244
	Recessive	AG+GG	278	392	1.00		1.00	
		AA	30	33	1.28 (0.76-2.15)	0.347	1.30 (0.77-2.19)	0.319
	Additive	------	------	------	1.16 (0.93-1.46)	0.191	1.17 (0.93-1.46)	0.185
rs72703442	Codominant	CC	209	298	1.00		1.00	
		AA	7	10	1.00 (0.37-2.67)	0.997	1.01 (0.38-2.70)	0.985
		AC	92	116	1.13 (0.82-1.57)	0.460	1.13 (0.81-1.56)	0.467
	Dominant	CC	209	298	1.00		1.00	
		AA+AC	99	126	1.12 (0.82-1.54)	0.483	1.12 (0.82-1.54)	0.487
	Recessive	AC+CC	301	414	1.00		1.00	
		AA	7	10	0.96 (0.36-2.56)	0.939	0.97 (0.37-2.59)	0.959
	Additive	------	------	------	1.09 (0.82-1.45)	0.546	1.09 (0.82-1.45)	0.545
rs2025327	Codominant	TT	231	338	1.00		1.00	
		CC	10	5	2.88 (0.97-8.55)	0.056	2.93 (0.99-8.69)	0.053
		CT	67	87	1.11 (0.77-1.59)	0.569	1.12 (0.78-1.60)	0.547
	Dominant	TT	231	338	1.00		1.00	
		CC+CT	87	92	1.21 (0.85-1.71)	0.288	1.22 (0.86-1.72)	0.271
	Recessive	CT+TT	298	420	1.00		1.00	
		CC	10	5	2.82 (0.95-8.33)	0.061	2.86 (0.97-8.45)	0.058
	Additive	------	------	------	1.26 (0.93-1.71)	0.131	1.27 (0.94-1.73)	0.121
rs55683539	Codominant	CC	176	248	1.00		1.00	
		TT	18	32	0.79 (0.43-1.46)	0.454	0.79 (0.43-1.46)	0.452
		TC	114	144	1.12 (0.82-1.53)	0.493	1.11 (0.81-1.52)	0.506
	Dominant	CC	176	248	1.00		1.00	
		TT+TC	132	176	1.06 (0.79-1.42)	0.715	1.05 (0.78-1.42)	0.730
	Recessive	TC+CC	290	392	1.00		1.00	
		TT	18	32	0.76 (0.42-1.38)	0.369	0.76 (0.42-1.38)	0.368
	Additive	------	------	------	0.99 (0.78-1.26)	0.939	0.99 (0.78-1.25)	0.927
rs2181559	Codominant	TT	121	183	1.00		1.00	
		AA	46	57	1.22 (0.78-1.92)	0.387	1.23 (0.78-1.93)	0.372
		AT	141	185	1.15 (0.84-1.58)	0.380	1.15 (0.83-1.57)	0.405
	Dominant	TT	121	183	1.00		1.00	
		AA+AT	187	242	1.17 (0.87-1.58)	0.306	1.16 (0.86-1.57)	0.318
	Recessive	AT+TT	262	368	1.00		1.00	
		AA	46	57	1.13 (0.75-1.72)	0.558	1.15 (0.75-1.75)	0.525
	Additive	------	------	------	1.12 (0.90-1.38)	0.307	1.12 (0.90-1.38)	0.304
rs10965059	Codominant	CC	249	277	1.00		1.00	
		TT	2	8	0.28 (0.06-1.32)	0.108	0.27 (0.06-1.28)	0.098
		TC	55	137	0.45 (0.31-0.64)	**<0.001**	**0.43 (0.30-0.62)**	**<0.001**
	Dominant	CC	249	277	1.00		1.00	
		TT+TC	57	145	0.44 (0.31-0.62)	**<0.001**	**0.42 (0.30-0.61)**	**<0.001**
	Recessive	TC+CC	304	414	1.00		1.00	
		TT	2	8	0.34 (0.07-1.62)	0.175	0.33 (0.07-1.59)	0.169
	Additive	------	------	------	0.46 (0.33-0.64)	**<0.001**	**0.45 (0.32-0.62)**	**<0.001**
rs10965064	Codominant	CC	128	171	1.00		1.00	
		GG	39	60	0.87 (0.55-1.38)	0.551	0.87 (0.54-1.38)	0.545
		GC	141	194	0.97 (0.71-1.33)	0.855	0.97 (0.71-1.33)	0.857
	Dominant	CC	128	171	1.00		1.00	
		GG+GC	180	254	0.95 (0.70-1.28)	0.719	0.95 (0.70-1.28)	0.719
	Recessive	GC+CC	269	365	1.00		1.00	
		GG	39	60	0.88 (0.57-1.36)	0.570	0.88 (0.57-1.36)	0.562
	Additive	------	------	------	0.94 (0.76-1.17)	0.588	0.94 (0.76-1.17)	0.584

SNP, single nucleotide polymorphism; OR, odds ratio; 95% CI, 95% confidence interval.

*p* values were calculated by logistic regression analysis.

Bold values indicate statistical significance (*p* < 0.05).

Age stratification showed that rs10965059 decreased the susceptibility to alcoholic ONFH individuals older than 40 years in the allele (*p*<0.001, OR=0.36), codominant (*p*<0.001, OR=0.45), dominant (*p*<0.001, OR=0.31), and additive models (*p*<0.001, OR=0.33, 9 **Table 4**). Rs10965064 only reduced the alcoholic ONFH susceptibility under the dominant model (*p*=0.049, OR=0.67).

**Table 4:** Relationships between MIR31HG SNPs and alcohol-induced ONFH susceptibility based on stratification by age.

SNP	Model	Genotype	> 40 years				≤ 40 years			
			Case	Control	OR (95% CI)	*p*	Case	Control	OR (95% CI)	*p*
rs10965059	Allele	C	340	380	1.00		213	311	1.00	
		T	34	106	**0.36 (0.24-0.54)**	**<0.001**	25	47	0.78 (0.46-1.30)	0.336
	Codominant	CC	154	142	1.00		95	135	1.00	
		TT	1	5	0.20 (0.02-1.73)	0.142	1	3	0.44 (0.04-4.41)	0.488
		TC	32	96	**0.31 (0.20-0.50)**	**<0.001**	23	41	0.80 (0.44-1.42)	0.440
	Dominant	CC	154	142	1.00		95	135	1.00	
		TT+TC	33	101	**0.31 (0.20-0.49)**	**<0.001**	24	44	0.77 (0.44-1.36)	0.369
	Recessive	TC+CC	186	238	1.00		118	176	1.00	
		TT	1	5	0.28 (0.03-2.40)	0.243	1	3	0.47 (0.05-4.62)	0.514
	Additive	—–	—–	—–	**0.33 (0.21-0.51)**	**<0.001**	—–	—–	0.77 (0.45-1.30)	0.323

SNP, single nucleotide polymorphism; OR, odds ratio; 95% CI, 95% confidence interval.

*p* values were calculated by logistic regression analysis.

Bold values indicate statistical significance (p < 0.05).

Besides, the necrotic sites stratification results (**Table 5**) indicated that rs10965059 played a protective role in alcoholic bilateral ONFH in the allele (OR=0.45, *p*<0.001), codominant (OR=0.40, *p*<0.001), dominant (OR=0.39, *p*<0.001), and recessive model (OR=0.42, *p*<0.001).

**Table 5:** Association between MIR31HG polymorphisms and ONFH risk stratified by necrotic sites.

SNP	Model	Genotype	OR (95% CI)	*p*
rs10965059	Allele	C	1.00	
		T	**0.45 (0.32-0.65)**	**<0.001**
	Codominant	CC	1.00	
		TT	0.34 (0.07-1.60)	0.171
		TC	**0.40 (0.27-0.59)**	**<0.001**
	Dominant	CC	1.00	
		TT+TC	**0.39 (0.27-0.58)**	**<0.001**
	Recessive	TC+CC	1.00	
		TT	0.43 (0.09-2.03)	0.284
	Additive	—–	**0.42 (0.29-0.61)**	**<0.001**

SNP, Single nucleotide polymorphism; OR, Odd ratios; CI, Confidence interval.

*p* values were calculated from logistic regression.

Bold values indicate statistical significance (p < 0.05).

### Haplotype analysis and MDR analysis

**Figure 1:**
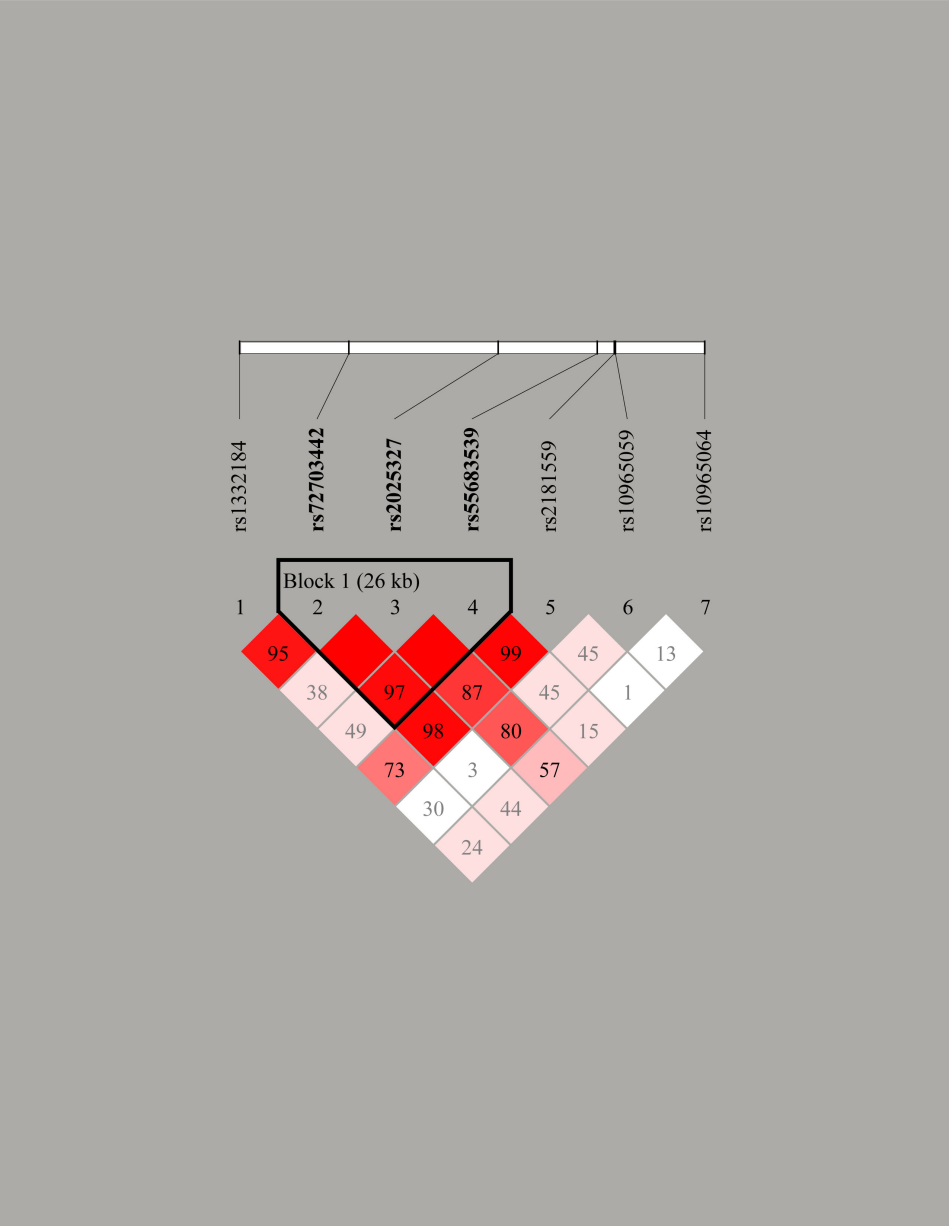
Linkage disequilibrium (LD) plots containing three polymorphisms from MIR31HG. Block 1 includes rs72703442, rs2025327, and rs55683539. The numbers inside the diamonds indicate the D’ for pairwise analyses.

We analyzed the haplotype of MIR31HG gene. **Table 6** represented that there was no linkage between haplotypes and alcoholic ONFH susceptibility (*p*>0.05). Besides, we found a LD block formed by rs72703442, rs2025327, and rs55683539 (**Figure 1**).

**Table 6:** Haplotype analysis of MIR31HG SNPs with alcohol-induced ONFH.

SNP	Haplotype	Frequency in cases	Frequency in controls	With adjustment		Without adjustment	
				OR (95% CI)	*p*	OR (95% CI)	*p*
rs72703442|rs2025327|rs55683539	ATT	0.171	0.157	1.11(0.84-1.48)	0.462	1.11(0.84-1.48)	0.464
rs72703442|rs2025327|rs55683539	CTT	0.073	0.087	0.83(0.57-1.21)	0.330	0.830.57-1.22)	0.345
rs72703442|rs2025327|rs55683539	CCC	0.140	0.114	1.26(0.93-1.71)	0.142	1.25(0.92-1.69)	0.153
rs72703442|rs2025327|rs55683539	CTC	0.384	0.362	1.10(0.89-1.35)	0.388	1.10(0.89-1.35)	0.393

SNP, Single nucleotide polymorphism; OR, Odd ratios; CI, Confidence interval.

**Figure 2:**
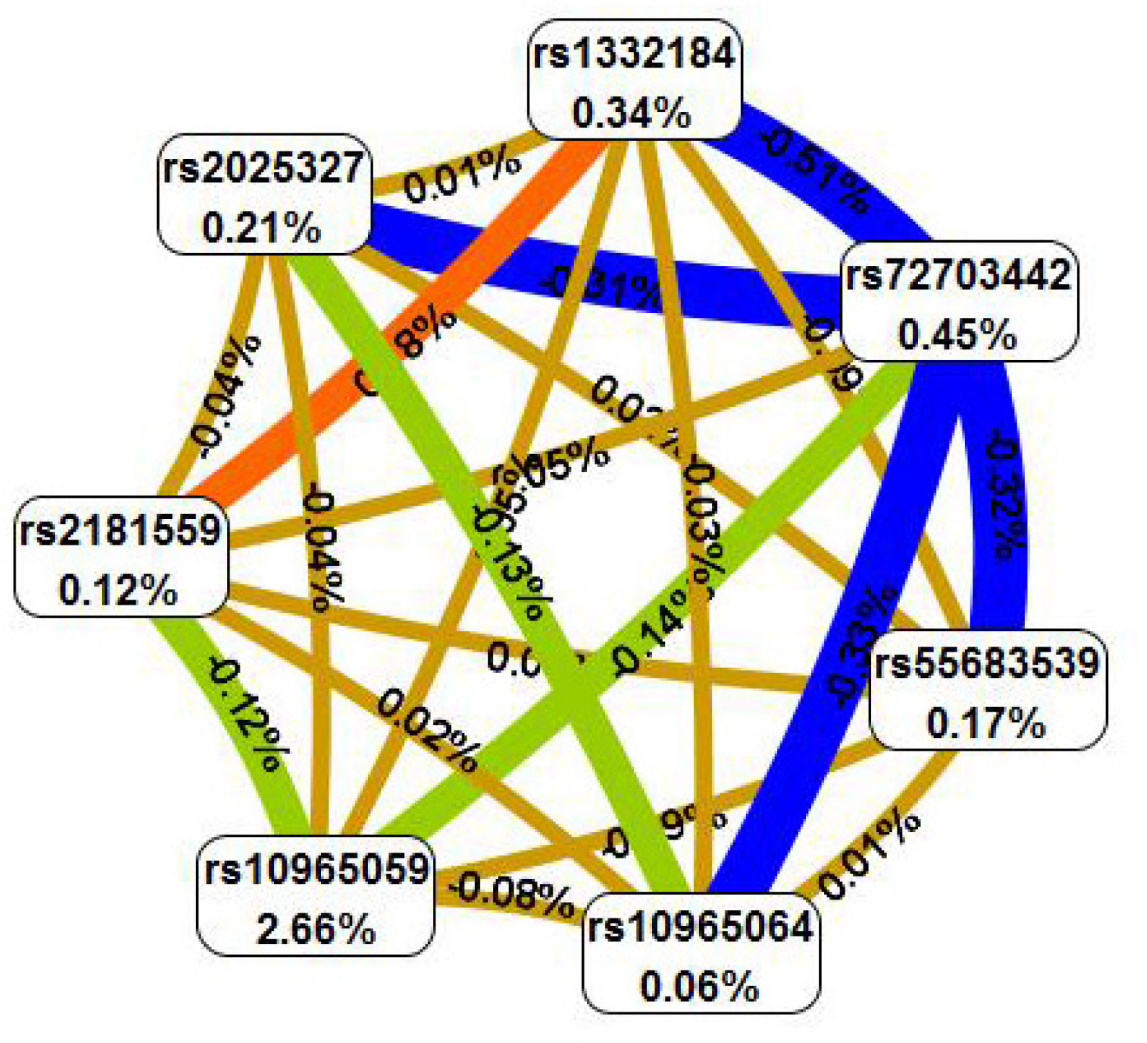
The Fruchterman-Reingold of SNP-SNP interactions. Each SNP is reported in per cent the value of Information Gain (IG), while numbers in the connections indicate the entropy-based IG for the SNP pairs. Orange bar indicate the high-level synergies on the phenotype, while the brown indicate a medium-level interaction, green and blue connections with negative IG values indicate redundancy or lack of synergistic interactions between the markers.

The Fruchterman-Rheingold of SNP-SNP interaction was presented in **Figure 2**. The results of **Supplemental Table 1** revealed that rs10965059 was the best single locus model to prediction the ONFH susceptibility (testing accuracy=0.581, CVC=10/10, *p* < 0.0001).

### FPRP analysis

As shown in **Supplemental Table 2**, all significant results of rs10965059 remained noteworthy, at the prior probability of 0.001 and FPRP threshold of 0.2.

## Discussion

We illustrated the relationship of MIR31HG polymorphisms with alcoholic ONFH susceptibility in this study. Results of our research indicated that MIR31HG-rs10965059 decreased the susceptibility of alcoholic ONFH overall. In the age stratification, we also observed that rs10965059 and rs10965064 had protective effect on alcoholic ONFH occurrence older than 40 years old. Besides, rs10965059 could reduce the alcoholic bilateral ONFH risk. These data underline the importance of MIR31HG in alcoholic ONFH occurrence and may serve as a new biomarker for the early prevention and treatment of alcoholic ONFH.

MIR31HG is located at the chromosomal locus 9q21.3 in humans. It has been described to participant in cell proliferation, differentiation in many diseases (20–22). In recent years, the functional role of MIR31HG in bone-related diseases has been studied. For example, Sun et al. found that MIR31HG was higher in tissues of osteosarcoma patients compared with healthy controls and it could regulate osteosarcoma cell growth and migration *via* miR-361 (23). Ma et al. have shown that MIR31HG was elevated in chordoma patients and MIR31HG silence repressed the migration, growth, and invasion of chordoma cells (24). Besides, suppression of MIR31HG could facilitate the differentiation of osteoblast in human adipose-derived stem cell (16). Moreover, a genome-wide association studies uncovered that MIR31HG polymorphism was related to radius bone density and content in children (25). These evidences led us to hypothesize that MIR31HG may have pathogenic significance in alcoholic ONFH. Here, we firstly observed the contribution of MIR31HG polymorphisms to alcoholic ONFH susceptibility. Rs10965059 exerted a protective role in alcoholic ONFH occurrence in both overall and stratified analysis.

Rs10965059 polymorphism is in the intron region of MIR31HG. Zhao et al. confirmed that the common intronic WDFY4 rs877819 affects the expression of WDFY4 gene by affecting YY1 binding (26). Choi et al. also found that intronic SNP (rs2280964) significantly correlated with reduced the expression of CXCR3 gene, which resulted in changes of immune cell responses to chemokine-cytokine signaling in ex vivo and vitro (27). Based on the above studies, we speculatedthat rs10965059 may affect the susceptibility of alcoholic ONFH by altering the expression of MIR31HG gene. In follow-up studies, we will explore the functional consequence of intronic SNP rs10965059 *in vitro* and ex vivo to support our hypothesis.

Inevitably, there are some limitations to this study. First, we did not conduct a functional analysis, which is essential to understand the role of MIR31HG in alcoholic ONFH. Second, the subjects were all Chinese Han population, and there may be a certain selection bias. Therefore, we needed animal or cell experiments and more ethnic groups to verify our findings.

## Conclusions

To sum up, we confirmed that MIR31HG variants have a significant correlation with the occurrence of alcoholic ONFH in a Chinese Han male population. This may provide new ideas for the prevention and diagnosis of alcoholic ONFH.

## Data availability statement

The data presented in the study are deposited in the Zenodo repository, accession number DOI: 10.5281/zenodo.7349781.

## Ethics statement

The studies involving human participants were reviewed and approved by Hainan Affiliated Hospital of Hainan Medical University. The patients/participants provided their written informed consent to participate in this study.

## Author contributions

JX designed this study protocol; WL drafted the manuscript; XW performed the DNA extraction and genotyping; JC performed the data analysis; FZ performed the sample collection and information recording. JX conceived and supervised the study. All authors read and approved the final manuscript.

## Funding

This study was supported by Hainan Provincial Natural Science Foundation of China (No. 822RC808) and Project supported by the Education Department of Hainan Province (No. Hnky2022ZD-13).

## Conflict of interest

The authors declare that the research was conducted in the absence of any commercial or financial relationships that could be construed as a potential conflict of interest.

## Publisher’s note

All claims expressed in this article are solely those of the authors and do not necessarily represent those of their affiliated organizations, or those of the publisher, the editors and the reviewers. Any product that may be evaluated in this article, or claim that may be made by its manufacturer, is not guaranteed or endorsed by the publisher.

## Abbreviations

ONFH, Osteonecrosis of the femoral head; OR, odds ratio; CI, confidence intervals; MAF, minor allele frequency; HWE, Hardy-Weinberg Equilibrium; CT, computed tomography; MRI, nuclear magnetic resonance imaging; LD, linkage disequilibrium.

## References

[1] Zalavras CG Lieberman JR. Osteonecrosis of the femoral head: evaluation and treatment. J Am Acad Orthopaedic Surgeons (2014) 22(7):455–64. doi: 10.5435/JAAOS-22-07-455 24966252

[2] Song Y Du Z Ren M Yang Q Wang Q Chen G. Association of gene variants of transcription factors PPARγ, RUNX2, osterix genes and COL2A1, IGFBP3 genes with the development of osteonecrosis of the femoral head in Chinese population. Bone (2017) 101:104–12. doi: 10.1016/j.bone.2017.05.002 28476574

[3] Hong G Han X He W Xu J Sun P Shen Y. Analysis of circulating microRNAs aberrantly expressed in alcohol-induced osteonecrosis of femoral head. Sci Rep (2019) 9(1):18926. doi: 10.1038/s41598-019-55188-6 31831773 PMC6908598

[4] Yoon BH Kim TY Shin IS Lee HY Lee YJ Koo KH. Alcohol intake and the risk of osteonecrosis of the femoral head in Japanese populations: a dose-response meta-analysis of case-control studies. Clin Rheumatol (2017) 36(11):2517–24. doi: 10.1007/s10067-017-3740-4 28685377

[5] Cui L Zhuang Q Lin J Jin J Zhang K Cao L. Multicentric epidemiologic study on six thousand three hundred and ninety five cases of femoral head osteonecrosis in China. Int Orthopaedics (2016) 40(2):267–76. doi: 10.1007/s00264-015-3061-7 26660727

[6] Guo Y Cao Y Gong S Zhang S Hou F Zhang X. Correlation analysis between CARMEN variants and alcohol-induced osteonecrosis of the femoral head in the Chinese population. BMC Musculoskeletal Disord (2020) 21(1):547. doi: 10.1186/s12891-020-03553-2 PMC7429464 32799824

[7] Wang J Shi X Yang H Du J Ouyang Y Wang H. Association between alcohol-induced osteonecrosis of femoral head and risk variants of MMPS in han population based on a case-control study. Oncotarget (2017) 8(38):64490–8. doi: 10.18632/oncotarget.16380 PMC5610020 28969088

[8] Liu C An F Cao Y Wang J Tian Y Wu H. Significant association between RETN genetic polymorphisms and alcohol-induced osteonecrosis of femoral head. Mol Genet Genomic Med (2019) 7(8):e822. doi: 10.1002/mgg3.822 31207150 PMC6687866

[9] Yang L Lin C Liu W Zhang J Ohgi KA Grinstein JD. ncRNA- and Pc2 methylation-dependent gene relocation between nuclear structures mediates gene activation programs. Cell (2011) 147(4):773–88. doi: 10.1016/j.cell.2011.08.054 PMC3297197 22078878

[10] Zhou J Yang L Zhong T Mueller M Men Y Zhang N. H19 lncRNA alters DNA methylation genome wide by regulating s-adenosylhomocysteine hydrolase. Nat Commun (2015) 6:10221. doi: 10.1038/ncomms10221 26687445 PMC4703905

[11] Tang S Xie Z Wang P Li J Wang S Liu W. LncRNA-OG promotes the osteogenic differentiation of bone marrow-derived mesenchymal stem cells under the regulation of hnRNPK. Stem Cells (Dayton Ohio). (2019) 37(2):270–83. doi: 10.1002/stem.2937 PMC7379496 30372559

[12] Liu C Cao Z Bai Y Dou C Gong X Liang M. LncRNA AK077216 promotes RANKL-induced osteoclastogenesis and bone resorption *via* NFATc1 by inhibition of NIP45. J Cell Physiol (2019) 234(2):1606–17. doi: 10.1002/jcp.27031 30132869

[13] Xiang S Li Z Weng X. The role of lncRNA RP11-154D6 in steroid-induced osteonecrosis of the femoral head through BMSC regulation. J Cell Biochem (2019) 120(10):18435–45. doi: 10.1002/jcb.29161 31190361

[14] Wang R Ma Z Feng L Yang Y Tan C Shi Q. LncRNA MIR31HG targets HIF1A and P21 to facilitate head and neck cancer cell proliferation and tumorigenesis by promoting cell-cycle progression. Mol cancer. (2018) 17(1):162. doi: 10.1186/s12943-018-0916-8 30458787 PMC6247607

[15] Nie FQ Ma S Xie M Liu YW De W Liu XH. Decreased long noncoding RNA MIR31HG is correlated with poor prognosis and contributes to cell proliferation in gastric cancer. Tumour Biol (2016) 37(6):7693–701. doi: 10.1007/s13277-015-4644-z 26692098

[16] Jin C Jia L Huang Y Zheng Y Du N Liu Y. Inhibition of lncRNA MIR31HG promotes osteogenic differentiation of human adipose-derived stem cells. Stem Cells (Dayton Ohio) (2016) 34(11):2707–20. doi: 10.1002/stem.2439 27334046

[17] Matsuo K Hirohata T Sugioka Y Ikeda M Fukuda A. Influence of alcohol intake, cigarette smoking, and occupational status on idiopathic osteonecrosis of the femoral head. Clin orthopaedics related Res (1988) 234):115–23. doi: 10.1097/00003086-198809000-00021 3409564

[18] Ficat RP. Idiopathic bone necrosis of the femoral head. early diagnosis and treatment. J Bone Joint Surg Br volume. (1985) 67(1):3–9. doi: 10.1302/0301-620X.67B1.3155745 3155745

[19] Wacholder S Chanock S Garcia-Closas M El Ghormli L Rothman N. Assessing the probability that a positive report is false: an approach for molecular epidemiology studies. J Natl Cancer Inst (2004) 96(6):434–42. doi: 10.1093/jnci/djh075 PMC7713993 15026468

[20] Gao J Chen F Hua M Guo J Nong Y Tang Q. Knockdown of lncRNA MIR31HG inhibits cell proliferation in human HaCaT keratinocytes. Biol Res (2018) 51(1):30. doi: 10.1186/s40659-018-0181-8 30180891 PMC6122774

[21] Yan S Tang Z Chen K Liu Y Yu G Chen Q. Long noncoding RNA MIR31HG inhibits hepatocellular carcinoma proliferation and metastasis by sponging microRNA-575 to modulate ST7L expression. J Exp Clin Cancer Res: CR (2018) 37(1):214. doi: 10.1186/s13046-018-0853-9 30176933 PMC6122648

[22] He A Chen Z Mei H Liu Y. Decreased expression of LncRNA MIR31HG in human bladder cancer. Cancer Biomarkers: Section Dis Markers (2016) 17(2):231–6. doi: 10.3233/CBM-160635 PMC13020480 27434291

[23] Sun Y Jia X Wang M Deng Y. Long noncoding RNA MIR31HG abrogates the availability of tumor suppressor microRNA-361 for the growth of osteosarcoma. Cancer Manage Res (2019) 11:8055–64. doi: 10.2147/CMAR.S214569 PMC6722458 31564967

[24] Ma X Qi S Duan Z Liao H Yang B Wang W. Long non-coding RNA LOC554202 modulates chordoma cell proliferation and invasion by recruiting EZH2 and regulating miR-31 expression. Cell Proliferation (2017) 50(6):e12388. doi: 10.1111/cpr.12388 28963737 PMC6529120

[25] Chesi A Mitchell JA Kalkwarf HJ Bradfield JP Lappe JM McCormack SE. A trans-ethnic genome-wide association study identifies gender-specific loci influencing pediatric aBMD and BMC at the distal radius. Hum Mol Genet (2015) 24(17):5053–9. doi: 10.1093/hmg/ddv210 PMC4527490 26041818

[26] Zhao H Yang W Qiu R Li J Xin Q Wang X. An intronic variant associated with systemic lupus erythematosus changes the binding affinity of Yinyang1 to downregulate WDFY4. Genes Immunity (2012) 13(7):536–42. doi: 10.1038/gene.2012.33 22972472

[27] Choi JW Park CS Hwang M Nam HY Chang HS Park SG. A common intronic variant of CXCR3 is functionally associated with gene expression levels and the polymorphic immune cell responses to stimuli. J Allergy Clin Immunol (2008) 122(6):1119–26.e7. doi: 10.1016/j.jaci.2008.09.026 18962861

