# Supplementary material for: The polymorphisms of MIR31HG gene is correlated with alcohol-induced osteonecrosis of the femoral head in Chinese Han male population
Source: Front Endocrinol (Lausanne). 2022 Nov 24;13:976165. doi: 10.3389/fendo.2022.976165 (PMC9731210; doi:10.3389/fendo.2022.976165)
Supplement: Supplementary file 1 [file DataSheet_1.docx]

Supplemental table 1 The primers for MIR31HG gene amplification and sequencing.

| **SNP** | **Forward Primer Sequence** | **Reverse Primer Sequence** | **UEP Sequence** |
| --- | --- | --- | --- |
| rs1332184 | ACGTTGGATGAAAGCACTGCTGGAAAGCCT | ACGTTGGATGGCTGCAGTTGCTTTCAATAA | atgaTGGAAAGCCTGAGCAGAGAAAT |
| rs72703442 | ACGTTGGATGAGGCAGCTTATAGGAATGGC | ACGTTGGATGGTCTTTGGTGGTGTTCTTCC | gggtAAGTGTCTTTACATTAGCAAGC |
| rs2025327 | ACGTTGGATGTAGTGTGTGACCTACATCTG | ACGTTGGATGATCCAAAATCTCAGCGGTAG | TGACCTACATCTGAGTTTC |
| rs55683539 | ACGTTGGATGTGGATCATCAGCAGTCCAG | ACGTTGGATGTCAGCCAGTTTAGCCACACT | TGAGAAAACAGTCATATCCT |
| rs2181559 | ACGTTGGATGGTTATGGGAAGAACTTGGTG | ACGTTGGATGCGTGAGACCTATTCACAAGC | gggGAACTTGGTGGGAGGT |
| rs10965059 | ACGTTGGATGTCTCACGGTTTTAAGAAGGG | ACGTTGGATGTGGCAGAAGGTGAAAGGCAA | gTCTTGTCTGCTGCCA |
| rs10965064 | ACGTTGGATGGAGTTCCACAGATATTCTGC | ACGTTGGATGGTAGTACTGGGACTCCAATC | AAGAGCAGAGACATAGAAATAG |

SNP, single nucleotide polymorphism; UEP, Unique.

Supplemental table 2 MDR analysis of SNP-SNP interaction

| Model | Training Bal. Acc. | Testing Bal. Acc. | CVC | OR (95% CI) | *p* |
| --- | --- | --- | --- | --- | --- |
| rs10965059 | 0.585 | 0.581 | 10/10 | 2.41 (1.67-3.49) | **<0.0001** |
| rs1332184,rs10965059 | 0.590 | 0.581 | 7/10 | 2.62 (1.79-3.83) | **<0.0001** |
| rs72703442,rs55683539,rs10965059 | 0.607 | 0.555 | 7/10 | 3.69 (2.41-5.65) | **<0.0001** |
| rs1332184,rs2181559,rs10965059,rs10965064 | 0.622 | 0.528 | 5/10 | 3.26 (2.25-4.72) | **<0.0001** |
| rs1332184,rs2025327,rs2181559,rs10965059,rs10965064 | 0.644 | 0.531 | 6/10 | 3.87 (2.67-5.60) | **<0.0001** |
| rs1332184,rs72703442,rs2025327,rs55683539,rs10965059,rs10965064 | 0.652 | 0.533 | 4/10 | 4.30 (2.95-6.27) | **<0.0001** |
| rs1332184,rs72703442,rs2025327,rs55683539,rs2181559,rs10965059,rs10965064 | 0.655 | 0.528 | 10/10 | 4.37(3.00-6.35) | **<0.0001** |

MDR, multifactor dimensionality reduction; Bal. Acc., balanced accuracy; CVC, cross–validation consistency; OR, odds ratio; CI, confidence interval.

*p* <0.05 indicates statistical significance.

Supplemental table 3 False positive report probability of the association *MIR31HG* rs10965059 and ONFH susceptibility in subgroups

| Model and variables | Genotype | OR (95% CI) | *p^a^* | Statistical power | Prior probability | | | | |
| --- | --- | --- | --- | --- | --- | --- | --- | --- | --- |
|  |  |  |  |  | 0.25 | 0.1 | 0.01 | 0.001 | 0.0001 |
| Overall analysis |  |  |  |  |  |  |  |  |  |
| rs10965059 T>C |  |  |  |  |  |  |  |  |  |
| Allele | T vs C | 0.48(0.35-0.66) | <0.001 | 0.401 | 0.000^b^ | 0.000 ^b^ | 0.002 ^b^ | 0.015 ^b^ | 0.135^b^ |
| Heterozygote | TC vs CC | 0.43 (0.30-0.62) | <0.001 | 0.210 | 0.000^b^ | 0.000 ^b^ | 0.003 ^b^ | 0.029 ^b^ | 0.227 |
| Dominant | TT+TC vs CC | 0.42 (0.30-0.61) | <0.001 | 0.180 | 0.000^b^ | 0.000 ^b^ | 0.003 ^b^ | 0.028 ^b^ | 0.225 |
| Additive | / | 0.45 (0.32-0.62) | <0.001 | 0.260 | 0.000^b^ | 0.000 ^b^ | 0.000 ^b^ | 0.004 ^b^ | 0.039^b^ |
| Stratification analysis |  |  |  |  |  |  |  |  |  |
| > 40 years |  |  |  |  |  |  |  |  |  |
| rs10965059 T>C |  |  |  |  |  |  |  |  |  |
| Allele | T vs C | 0.36(0.24-0.54) | <0.001 | 0.056 | 0.000^b^ | 0.000 ^b^ | 0.001 ^b^ | 0.014 ^b^ | 0.123^b^ |
| Heterozygote | TC vs CC | 0.31(0.20-0.50) | <0.001 | 0.025 | 0.000^b^ | 0.001 ^b^ | 0.006 ^b^ | 0.059 ^b^ | 0.386 |
| Dominant | TT+TC vs CC | 0.31(0.20-0.49) | <0.001 | 0.020 | 0.000^b^ | 0.001 ^b^ | 0.003 ^b^ | 0.026^b^ | 0.208 |
| Additive | / | 0.33(0.21-0.51) | <0.001 | 0.031 | 0.000^b^ | 0.001 ^b^ | 0.002 ^b^ | 0.019^b^ | 0.163 ^b^ |
| Necrotic sites |  |  |  |  |  |  |  |  |  |
| rs10965059 T>C |  |  |  |  |  |  |  |  |  |
| Allele | T vs C | 0.45(0.32-0.65) | <0.001 | 0.287 | 0.000^b^ | 0.001^b^ | 0.007 ^b^ | 0.067 ^b^ | 0.420 |
| Heterozygote | TC vs CC | 0.40(0.27-0.59) | <0.001 | 0.130 | 0.000^b^ | 0.001^b^ | 0.003 ^b^ | 0.028 ^b^ | 0.227 |
| Dominant | TT+TC vs CC | 0.39(0.27-0.58) | <0.001 | 0.110 | 0.000^b^ | 0.000^b^ | 0.003 ^b^ | 0.029 ^b^ | 0.232 |
| Additive | / | 0.42(0.29-0.61) | <0.001 | 0.180 | 0.000^b^ | 0.001^b^ | 0.003 ^b^ | 0.028^b^ | 0.225 |

Abbreviations: CHD, coronary heart disease; OR: odds ratio; CI, confidence interval.

*p^a^ <0.05* indicates statistical significance.

^b^ The level of false positive report probability threshold was set at 0.2 and noteworthy findings are presented.
